# Implication of combined PD-L1/PD-1 blockade with cytokine-induced killer cells as a synergistic immunotherapy for gastrointestinal cancer

**DOI:** 10.18632/oncotarget.7243

**Published:** 2016-02-08

**Authors:** Congqi Dai, Fengjuan Lin, Ruixuan Geng, Xiaoxiao Ge, Wenbo Tang, Jinjia Chang, Zheng Wu, Xinyang Liu, Ying Lin, Zhe Zhang, Jin Li

**Affiliations:** ^1^ Department of Medical Oncology, Fudan University Shanghai Cancer Center; Department of Oncology, Shanghai Medical College, Fudan University, Shanghai, China; ^2^ Department of Oncology, Tongji University Tianyou Hospital, Shanghai, China

**Keywords:** PD-L1/PD-1, CIK, NKG2D, immunotherapy, gastrointestinal tumor

## Abstract

Cytokine-induced killer (CIK) cells represent a realistic approach in cancer immunotherapy with confirmed survival benefits in the context of metastatic solid tumors. However, therapeutic effects are limited to a fraction of patients. In this study, immune-resistance elements and ideal combination therapies were explored. Initially, phenotypic analysis was performed to document CD3, CD56, NKG2D, DNAM-1, PD-L1, PD-1, CTLA-4, TIM-3, 2B4, and LAG-3 on CIK cells. Upon engagement of CIK cells with the tumor cells, expression of PD-1 on CIK cells and PD-L1 on both cells were up-regulated. Over-expression of PD-L1 levels on tumor cells via lentiviral transduction inhibited tumoricidal activity of CIK cells, and neutralizing of PD-L1/PD-1 signaling axis could enhance their tumor-killing effect. Conversely, blockade of NKG2D, a major activating receptor of CIK cells, largely caused dysfunction of CIK cells. Functional study showed an increase of NKG2D levels along with PD-L1/PD-1 blockade in the presence of other immune effector molecule secretion. Additionally, combined therapy of CIK infusion and PD-L1/PD-1 blockade caused a delay of *in vivo* tumor growth and exhibited a survival advantage over untreated mice. These results provide a preclinical proof-of-concept for simultaneous PD-L1/PD-1 pathways blockade along with CIK infusion as a novel immunotherapy for unresectable cancers.

## INTRODUCTION

In spite of significant advances in diagnosis and treatment, gastrointestinal cancers are still mostly unresectable at an advanced stage, and traditional therapeutic options are always limited. Given that, other alternative effective strategies are required to provide a survival benefit [1]. Cancer immunotherapy, especially adoptive cell therapy (ACT), represents an effective therapy strategy in the setting of solid tumors that are unmanageable using standard treatments, and holds great promise as an emerging possibility involved in the multidisciplinary treatment plans [2, 3]. However, in clinical practice, some crucial gaps including generation of efficient number of lymphocytes *in vitro*, HLA-restriction barriers, and the safety issues in application are needed to be bridged [4, 5].

Cytokine-induced killer (CIK) cells, possessing an advantageous profile of easy production *in vitro*, MHC-unrestricted cytotoxicity, and low graft-versus-host disease (GVHD) risk, are reported to exert a potent immune response against both solid and hematologic malignances [6-10]. They are heterogeneous lymphocytes activated and expanded *in vitro*, and carry out their cytotoxic potential mainly through recognition of NKG2D rather than T cell receptor (TCR) [11, 12]. NKG2D is an activating receptor, serving the role of detection and elimination for tumor cells via interaction with its ligands, MHC class I-related molecules A and B (MIC A/B), and six members of the unique long 16-binding proteins (ULBPs), which are exclusively up-regulated on the surface of malignant cells [13-15]. Although the precise mechanism underlying tumor-killing by CIK cells has yet to be completely clarified, CIK adoptive transfer in clinical trials demonstrated a significant improvement in outcomes [16-20], offering a pragmatic treatment option for patients with advanced cancers. Nonetheless, therapeutic benefits of CIK cells are often limited to a fraction of patients [20]. An identification of potential immune-resistance mechanism and development of novel therapeutic strategies are required. Several immune checkpoints, such as PD-L1 and PD-1, have been implicated in tumor immunoevasion in many human malignancies. Exploring combinational approaches using checkpoint blockade to enhance antitumor effects of ACT might be beneficial to maximize treatment efficacy.

Programmed cell death 1 (PD-1), a 288-amino-acid protein, is mainly expressed on the activated T/B cells, and NK cells [21, 22], although the studies on its biological activity have been mostly focused on T cells. PD-1 has been found to mediate inhibitory signals through interaction with its ligands, PD-L1 (B7-H1) and PD-L2 (B7-DC), mainly expressed and up-regulated on a wide variety of malignant cells, dampening antitumor immunities in the experimental and clinical researches [23-27].

Combinational approaches to enhancing CIK-cytolytic activity against cancer are considered in the scenario: 1. the proven safety and efficient tumoricidal features of CIK cells allow investigation with other potential synergistic and complementary strategies; 2. given their T-cell origin, it is reasonable to study a role for PD-L1/PD-1 signaling in adoptive cell immunotherapy using CIK cells. In the study, we presented new evidence that PD-L1 expression suppresses tumor-killing activity of CIK cells against allogeneic tumor cell lines, and PD-L1/PD-1 blockade strengthens tumoricidal activity of CIK cells, providing preclinical data for simultaneous PD-L1/PD-1 blockade along with CIK infusion as a novel treatment approach for gastrointestinal cancer patients.

## RESULTS

### *In vitro* expansion and phenotypic characteristics of CIK cells

**Figure 1:**
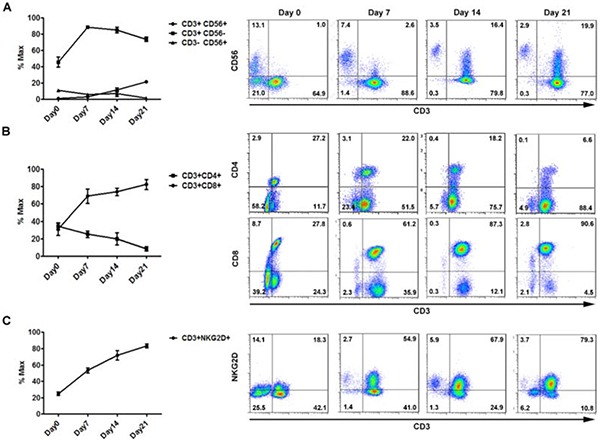
*In vitro* expansion and main phenotypes of CIK cells derived from donors (*n* = 10) Differential expression of three main phenotypic subsets of CIK cells over *in vitro* culture are shown for identification of CIK cells, CD3/CD56 **(A),** T cell associated immune receptors, CD3/CD4/CD8 **(B),** and major activating receptors of CIK cells, CD3/NKG2D **(C).** Flow cytometric analysis was performed over expansion periods on day0, day7, day14, and day21. Each representative picture was accordingly shown on the right panel.

At the end of expansion prior to injection, the majority of CIK cells has the CD3+CD56− phenotype, with a median percentage on total CIK cells of 73.4% (range, 68.5%-77.9%). Notably, the subset of NKT cells co-expressing CD3 and CD56 (CD3+CD56+) increased over the culture time, from 0.9% (range, 0.4%-1.48%) at day 0, to 2.77% (range, 1.8%-3.8%) at day 7, 11.36% (range, 7.2%-20.8%) at day 14, 21.33% (range, 16.4%-23.2%) at day 21 (Figure 1A). In addition, the proportion of CD3+CD8+CIK cells increased from 30.8±6.8% at day 0 to 82.1±5.7% at day 21, in contrast with those co-expressing CD3 and CD4 molecules that decreased from 34.4±4.2% at day 0 to 8.2±2.6% at day 21 (Figure 1B). The majority of CIK cells expressed the activating receptor, NKG2D, mainly responsible for CIK target recognition, and the percentage increased from 24.9±2.2% at day 0 to 83.2±2.4% at day 21 (Figure 1C). At the end of culture, CIK-immune pattern was observed for other potential immune-associated markers, including DNAM-1, LAG-3, and CTLA-4 which were 80.7±1.1%, 35.4±5.8%, and 25.2±1.4%, respectively, as well as 2B4 that was rarely detected for CIK cells (Supplementary Figure S1).

### PD-L1 expression on tumor cells suppresses cytotoxicity of CIK cells

**Figure 2:**
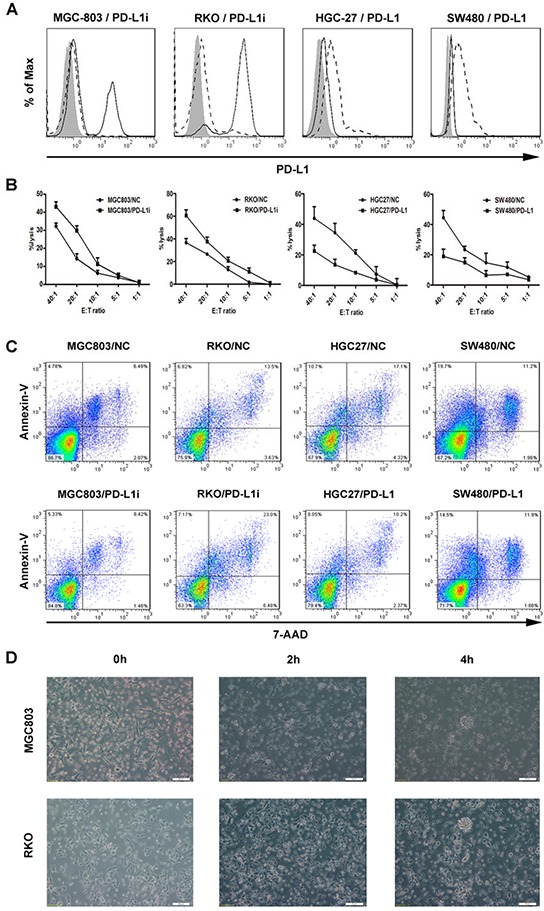
Stable variations in PD-L1 expression on the tumor cells via lentiviral transduction and corresponding influences upon tumoricidal activity of CIK cells **A.** Gastric and colorectal cancer cell lines (MGC803 and RKO) were transduced with lentiviral vectors containing siRNA directed against PD-L1, whereas HGC27 and SW480 were transfected with PD-L1 cDNA. After 72 hours, transfected tumor cells were confirmed for PD-L1 expression by flow cytometry. *filled* isotype staining; *bold line* PD-L1-stained untransfected cell lines; *dashed line* PD-L1-stained transfected cell lines with siRNA (MGC803 and RKO) or PD-L1 cDNA (HGC27 and SW480). **B.** CIK cells exerting cytotoxicity against the tumor cells were assessed by a non-radioactive cytotoxicity assay. Comparative curves were drawn between the tumor cells transfected with siRNA or PD-L1 cDNA and with scrambled shRNA as negative control group. Results represent at least three independent experiments using CIK cells from the same donor and are shown as Mean±SEM. **C.** CIK cells was pre-incubated with above tumor cells in 2ml culture medium per well in a 6-well-plate at a E:T ratio of 10:1 overnight, and both suspending and adherent cells were harvested and analyzed for cell apoptosis assay. **D.** Concordantly, obvious lysis of representative tumor cells (MGC803 and RKO) was observed over time by microscopy.

Over-expression of PD-L1 on the tumor cells has been found to impair antitumor immunity [28, 29]. To test the functional effects of PD-L1 expression on the malignant cells, we adopted the tool of lentivirus transduction that can achieve stable knockdown and over-expression of PD-L1. Initially, two panels of tumor cell lines, gastric cancer cells (HGC27, MNK45, SNU216, SGC7901, and MGC803) and colorectal cancer cells (SW480, HT-29, RKO, and HCT116) were screened at the mRNA and protein levels for the detection of their constitutive expression of PD-L1 molecule (Supplementary Figure S2A). HGC27 and SW480 displaying the lowest PD-L1 levels in contrast with MGC803 and RKO with the highest were selected from each panel, respectively, and defined as the target tumor cells to optimize the experimental efficiency (Supplementary Figure S2B). Using a non-radioactive cytotoxicity assay, we showed a significant enhancement in the CIK-cytolytic activity against MGC803 or RKO that was each transduced with lentiviral vectors containing siRNA directed against PD-L1, whereas CIK cells exerted impaired cytotoxicity against HGC27 or SW480 that were transfected with PD-L1 cDNA, in an Effect:Target (E:T) ratio-dependent manner (Figure 2A and 2B). In addition, both MGC803 and RKO whose PD-L1 expression were knockdown exhibited aggravated apoptosis, compared to negative control(NC) groups, whereas HGC27 and SW480 that were characterized with PD-L1 overexpression underwent attenuated apoptotic effects induced by CIK engagement, at the E:T ratio of 10:1 (Figure 2C). Meanwhile, obvious lysis of the tumor cells was correspondingly observed over time under the microscope, and the structure of cells was distorted with fuzzy membrane, along with a majority of wall-attached tumor cells turning to suspend in the medium after 4 hour co-culture. (Figure 2D).

### PD-L1/PD-1 blockade augments CIK cytotoxicity against gastric and colorectal cancer cells

**Figure 3:**
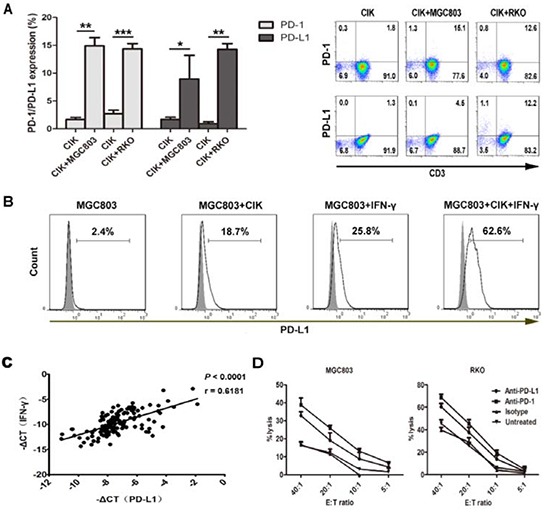
PD-L1/PD-1 pathway blockade efficiently increases tumor-killing activity of CIK cells **A.** The levels of PD-1 and PD-L1 on the CIK cells were observed after 12 hours co-culture with MGC803 or RKO. Data from three independent experiments were shown as Mean±SEM, and statistical significance was determined by Student t test (* means *P*<0.05, ** means *P*<0.01, and *** means *P*<0.001). Representative pictures are indicated on the right panel. **B.** PD-L1 levels on the tumor cells (MGC803) were detected by the co-culture, as well as those with the adding of IFN-γ (200U/ml). *filled* isotype staining; *bold line* PD-L1-stained MGC803. **C.** Correlation was performed between IFN-γ and PD-L1 mRNA levels in the gastric cancer tissues. Pearson's r and *P* value are displayed. -ΔCt indicates the difference in the threshold cycle between the target genes and β-actin. **D.** CIK cells exerting cytotoxicity against MGC803 or RKO, respectively, were assessed by a non-radioactive cytotoxicity assay, with or without PD-L1/PD-1 blockade using indicated antibodies (20μg/ml). Corresponding isotype antibody (20μg/ml) for PD-L1/PD-1 was used as a control group. Results represent at least three independent experiments using the CIK cells from the same donor and are shown as Mean±SEM.

Since PD-1 has been reported to be practically absent on naïve T lymphocytes and up-regulated upon activation [30], we intended to observe whether CIK cells have the same phenomenon by modeling the co-incubation of CIK cells with tumor cells (MGC803 and RKO), and revealed that the levels of both PD-1 and PD-L1 on the CIK cells increased significantly, either after 12 hour-co-culture (Figure 3A), or for a period of 24 hours (Supplementary Figure S3); the PD-L1 levels on the tumor cells also showed a significant elevation upon the co-culture, and the tendency was further increased with the adding of IFN-γ (Figure 3B). Remarkably, the detection of 136 clinical specimens from patients with gastric cancer indicated that mRNA levels of IFN-γ in the gastric cancer tissues positively correlated with PD-L1 (*P*<0.0001, r=0.6181) (Figure 3C). Concordant with proofs concerning the involvement of PD-L1 in inducing immune evasion by tumors [28-32], we further revealed that using blocking PD-L1/PD-1 antibodies both resulted in an increased tumoricidal ability of CIK cells against MGC803 and RKO, in an E:T ratio-dependent manner (Figure 3D).

**Figure 4:**
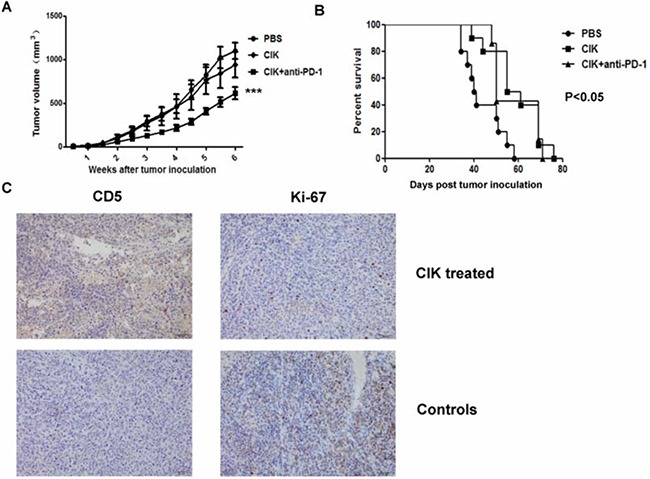
*In vivo* cytotoxicity of CIK cells against murine gastric cancer model Nude mice were subcutaneously implanted with an 8mm^3^ tumor fragment prepared beforehand with MGC803 cells injected into 5 mice. Three days after tumor implantation, mice received weekly intravenous infusions of 10^7^ CIK cells. Anti-PD-1 antibody was pre-added to block PD-1 on CIK cells for 1 hour. **A.** An obvious tumor growth delay was observed in combined CIK with anti-PD-1-treated mice compared with the control group (*P*<0.001), whereas no significant difference was found in CIK alone-treated mice (*P*>0.05). **B.** Percentage survival of MGC803 tumor-bearing mice treated with CIK cells with or without anti-PD-1 antibody was significantly increased compared with untreated mice using Kaplan-Meier analysis (*P*<0.05), whereas no significant difference was found between CIK cells-treated groups with or without anti-PD-1 antibody (*P*>0.05). **C.** At the end of infusion, representative pictures of CIK cells infiltration at tumor sites were shown by IHC using antibodies against CD5, and expression of ki-67 was shown in tumors from CIK-treated mice and control, scale bar, 50μm. Tumor growth and survival data were shown independently with 6 to 10 mice per group.

Furthermore, we tested *in vivo* cytotoxicity of CIK cells against tumor xenografts in nude mice. There was a significant reduction of tumor size in combined CIK cells and anti-PD-1-treated mice compared with untreated controls (*P*<0.001), whereas tumor delay in CIK cells alone-treated mice did not show a significant difference when comparing to controls (*P*>0.05) (Figure 4A). Besides, treatment of mice with CIK cells with or without anti-PD-1 antibody could both led to a better survival outcome than untreated controls (*P*<0.05) (Figure 4B), whereas no significant difference was observed between CIK cells-treated groups with or without anti-PD-1 antibody (*P*>0.05). At the end of infusion, we confirmed the presence of CIK cells infiltration at tumor sites, and expression of ki-67 was shown as reduced in tumors from CIK-treated mice (Figure 4C).

### Neutralizing of NKG2D interactions with its ligands exerts impaired cytotoxic activity of CIK cells

**Figure 5:**
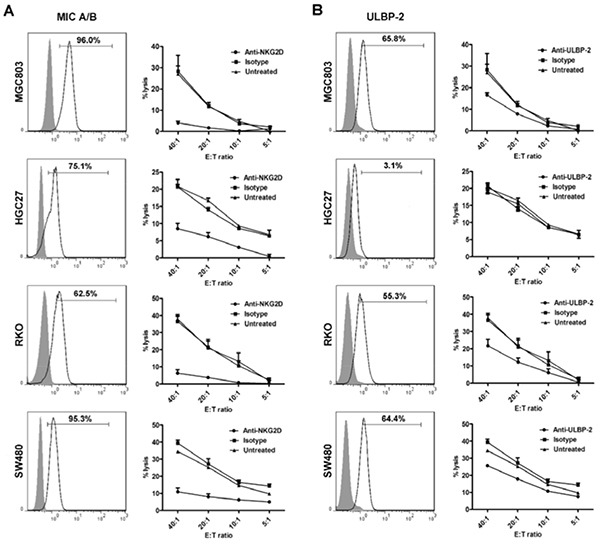
Blocking of NKG2D binding with its ligands results in impaired cytotoxic activity of CIK cells against tumor cell lines (MGC803, HGC27, RKO, and SW480) **A.** All the tumor cell lines were observed for MIC A/B expression. Besides, the lytic activity of CIK cells, with or without blocking its recognition receptor, NKG2D, was assessed by cytotoxicity assay. **B.** Meanwhile, all the above cell lines were detected for the levels of ULBP-2, another crucial ligand for NKG2D. Antibody-blockade of ULBP-2 was conducted to observe cytotoxicity of CIK cells against all the above cells that differentially express ULBP-2. *filled* isotype staining; *bold line* MIC A/B− or ULBP-2-stained tumor cell. All the data on the curves represent three independent experiments using CIK cells from the same donor and are shown as Mean±SEM.

It has been previously proven that NKG2D, involved in the target recognition of CIK cells, mainly binds with the non-classical MHC molecules, MIC A/B and ULBP-2 [10, 33]. Hence, SW480, HGC27, RKO, and MGC803 cell lines were analyzed for NKG2D ligands expression. Except for HGC27 that mostly expresses MIC A/B (75.1%) but very low positive ratio of ULBP-2 (3.1%), all tumor cells expressed high levels of both molecules (Figure 5A and 5B). Thus unlike the results that blocking the NKG2D receptor dramatically dampened the lytic activity of CIK cells against all the tumor cells, with significant differences at the E:T ratios of 40:1 (*P*<0.001), 20:1 (*P*<0.001), 10:1 (*P*<0.01), and 5:1 (*P*<0.01) (with HGC27 for example), antibody-blockade of ULBP-2 could merely induced dysfunction of CIK cells against the cells, including SW480, RKO, and MGC803 that express high levels of ULBP-2, rather than HGC27. (Figure 5A and 5B).

### Increasement of NKG2D receptor in the enhancement of CIK cytotoxicity by PD-L1/PD-1 pathway blockade

**Figure 6:**
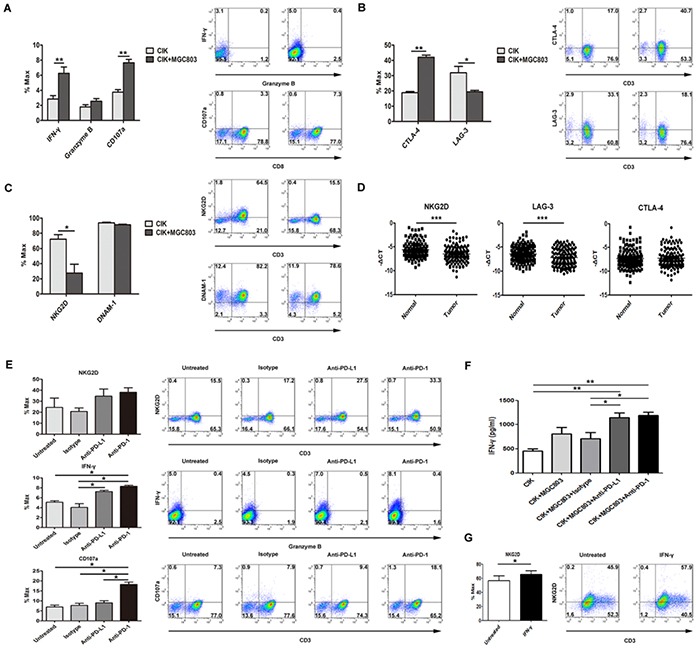
Disrupting PD-L1/PD-1 binding using blocking antibodies markedly induced an increase in multiple immune effector molecules **A.** IFN-γ and CD107a, as direct measurements of cytotoxicity of CIK cells against tumor cells, as well as granzyme B, were observed by the co-incubation at E:T ratio of 5:1, along with the detection for a potentially responsive phenotype in terms of the co-inhibitory molecules, CTLA-4 and LAG-3 **(B),** and the activating receptors, NKG2D and DNAM-1 **(C). D.** Gastric clinical specimens were examined for analyzing the difference in the mRNA levels of these functional receptors between the gastric cancer samples and matched adjacent normal samples, and statistical significance was determined by paired t test. **E.** Associated immune-promoting molecules, IFN-γ and CD107a, accompanied with NKG2D levels, were examined after PD-L1/PD-1 pathway blockade using indicated antibodies (20μg/ml), among the test groups and the control groups (indicated as untreated group and isotype antibody group). **F.** 5×10^5^ CIK cells were co-cultured in triplicate with 1×10^5^ tumor cells in 500 μl culture medium per well in a 48-well-plate. Anti-PD-L1 and anti-PD-1 together with isotype antibody (20μg/ml) were separately added into each well. After 48 h, the levels of IFN-γ in the supernatants were evaluated by ELISA. **G.** Using the same co-culture condition described above, changes of NKG2D levels were observed with the direct adding of IFN-γ cytokine. Results represent three independent experiments and are shown as Mean±SEM (* means *P*<0.05, ** means *P*<0.01, and *** means *P*<0.001).

To study possible variations of associated immune markers and cytokines involved in CIK cells killing against tumor cells, with MGC803 for assessment, we detected that IFN-γ and CD107a, as direct measurements of cytotoxicity of CIK cells, were induced by the co-incubation, whereas no significant change was observed on granzyme B secretion (Figure 6A). CIK cells also showed a functionally responsive phenotype in terms of other inhibitory markers, CTLA-4 and LAG-3 (Figure 6B), rather than 2B4 and Foxp3 (data not shown). In addition, the levels of activating receptor NKG2D on CIK cells displayed a marked reduction upon engagement with tumor cells, whereas the declining degree of DNAM-1 was negligible (Figure 6C). More intriguingly, the induced expression changes of these immune-modulatory receptors including NKG2D, LAG-3, and CTLA-4 on the CIK cells when engaged with the tumor cells like MGC803 cells, were largely consistent with the differences between gastric tumor tissues and matched adjacent normal samples observed at the mRNA levels in examined specimens (Figure 6D). Additionally, disrupting PD-L1/PD-1 binding using blocking antibodies could release a substantial elevation of associated immune-promoting molecules, IFN-γ and CD107a, accompanied with an increase of NKG2D levels (Figure 6E), while dampening the inhibitory signaling mediated by CTLA-4 and LAG-3 (Supplementary Figure S4), although without reaching statistical significance. Apart from detection of intracellular IFN-γ levels of CIK cells, IFN-γ cytokine release assay further verify the increase in tumor-killing ability of CIK cells by blocking PD-L1/PD-1 binding (Figure 6F). Otherwise, by directly adding cytokine IFN-γ into the culture medium, NKG2D levels on the CIK cells were significantly up-regulated (Figure 6G).

## DISCUSSION

The present study reports the effective killing activity of CIK cells against allogeneic gastric and colorectal cancer cell lines, and for the first time reflects the potentialities of PD-L1/PD-1 blockade to enhance tumoricidal activity of CIK cells as a promising adoptive immunotherapy, and presents crucial immunologic elements involved in the complex interactions between tumor cells and immune effectors in the preclinical model of CIK cells, with insights on their prospective for clinical translation.

In the study, an easy availability of CIK cells obtained from PBMCs was shown after expansion *in vitro* for 14-21 days, by sequentially adding IFN-γ, OKT-3, and IL-2. Previous studies have shown that CIK cells represented dual properties of T and NK cells [34], as consistent with present study by positive expression of immune markers, CD3, CD4, CD8, and CD56, and functional phenotypes, NKG2D and DNAM-1, two main activating receptors mediating the cytotoxicity of CIK cells [35]. To investigate the elements that might hamper the effectiveness of adoptive cell immunotherapy using CIK cells, and to achieve optimized destruction of malignancy within tumor microenvironment, several immune inhibitory checkpoints, CTLA-4, 2B4, LAG-3, and PD-L1/PD-1 presumably correlated with a severe exhausted phenotype of T cells [36-38], were documented on the CIK cells. The results revealed that via lentivirus vector delivery that varies PD-L1 expression on the tumor cells, CIK-mediated antitumor activity could be inhibited by the PD-L1 over-expression on the tumor cells. In addition, it was found that the PD-1 and PD-L1 expression on CIK cells and PD-L1 on tumor cells were inducible in response to the co-incubation. This feedback effect was considered to counterbalance the effector function of CIK cells against tumor, and it could be verified by the reverse proof that PD-L1/PD-1 signaling blockade using blocking antibodies enhanced CIK-cytotoxic activity. Otherwise, the analysis on clinical gastric cancer tissues by qRT-PCR showed PD-L1 correlated positively with IFN-γ level, reconfirming that the PD-L1 expression on malignant cells could be directly induced by IFN-γ secreted by immune cells [30-32]. Altogether, we hypothesize that IFN-γ might be secreted not merely to mount a potent immunity against tumor, and meanwhile induces an immune resistance, such as PD-L1/PD-1 inhibitory signaling, to protect from exorbitant immune reaction, however, they are often wittingly coopted by cancer cells to evade host immunity [15, 38]. A schematic diagram was drawn to illustrate the relationship between CIK cells and tumor cells (Supplementary Figure S5).

It has been confirmed that the mechanism underlying tumor recognition by CIK cells were mainly through the interaction of NKG2D with its ligands on the tumor cells, whereas other activating natural cytotoxicity receptors, NKp30, NKp44, NKp46 and killer inhibitory receptors NKG2A and CD94 were rarely involved in the CIK-induced cytolysis effects [10, 35]. NK cells and γδT cells were reported to recognize and kill the cells that express NKG2D ligands in a process called human lymphoid stress surveillance [39], and the ligands for NKG2D were poorly expressed on normal tissues while up-regulated on the transformed cells [14]. In the study, antibody blocking experiments showed impediment of NKG2D receptor or its ligand ULBP-2 led to a marked decrease in the CIK-cytotoxic function, relying upon their constitutive expression levels. However, although the cytotoxicity of CIK cells were largely reduced by the blocking of NKG2D-ligands binding, it could not completely abrogate their lytic effects against tumor cells, suggesting the role of NKG2D interaction with its ligands was a main but non-exclusive mechanism mediating cytotoxic effects of CIK cells, and other molecules might be implicated. The aim of this study was not to dig out in detail mechanisms underlying the cytotoxicity of CIK cells, however, to investigate a more complete definition of tumor-specific ligands and their functional mechanisms is of great value for identifying personalized and biological features of cancer patients as therapeutic targets and needs to be further explored.

Moreover, the critical role of NKG2D-mediated tumor surveillance are supported by the proof that NK and CD8+ T cells could rapidly eradicate the tumor cells that were transfected with NKG2D ligands and injected into mice [40], and many clinical retrospective observations have presented the prognostic value of NKG2D ligands in different cancer types and they might be viable targets for developing effective cancer immunotherapy [41, 42]. Additionally, decreased NKG2D expression on immune cells is involved in immune evasion [43, 44], and CIK cells also displayed a remarkable reduction of NKG2D expression upon engagement with tumor cells *in vitro*. Our results revealed that PD-L1/PD-1 signaling blockade could induce an elevation of NKG2D expression levels, and this increase might be a concomitant consequence with the release of multiple immune-promoting molecules, such as IFN-γ that we have shown to directly stimulate the levels of NKG2D on CIK cells, which is compatible with the previous study demonstrating that NKG2D molecule could be modulated by various cytokines and tumor-derived factors [45], and with clinical specimen analysis showing NKG2D expression positively correlated with IFN-γ level (Supplementary Figure S6).

In the study, we acknowledge that allogeneic cell lines were used to observe the tumoricidal role of CIK cells, rather than autologous cell lines as the optimal tool to evaluate patient-specific biologic immunities, due to the restriction caused by a lack of specimens from patients with unresectable tumors. However, considering the scenario that patients were infused with CIK cells derived from their relatives, husbands or their children due to low proliferating efficiency by autologous culture, and were all well tolerated with no adverse reactions except for a short-term fever after infusion within the first day, allogeneic CIK cells might be proper alternatives for a population like that. In addition, we showed a low alloreactivity of CIK cells against allogeneic peripheral blood mononuclear cells and there were no obvious differences in lytic activity between different E:T ratios (Supplementary Figure S7). Indeed, both autologous and allogeneic CIK cells have proven effective in clinical trials for the treatment of different malignancies [16-20].

Consistent with a previous finding showing CIK cells could rapidly migrate into the tumor sites after infusion, in particular the sites expressing NKG2D ligands, and spare normal tissues [46], CIK cells have been demonstrated in our study to infiltrate into the tumor sites in mice model, and no discomfort of the mice was observed during the whole therapy. Additionally, the therapeutic strategy of CIK cell adoptive transfer with PD-L1/PD-1 blockade could cause a delay of *in vivo* tumor growth and display a survival advantage over untreated controls.

Considering all the above advantages of CIK cells and the synergistic effects led by PD-L1/PD-1 blockade, combined therapy of simultaneous CIK infusion and PD-L1/PD-1 signaling blockade should be put forward as a novel promising candidate for clinical trial, especially in the setting of unresectable metastatic cancers.

## MATERIALS AND METHODS

### *In vitro* generation and phenotypic detection of CIK cells

Ten to fifteen milliliters of heparinized peripheral blood were collected for CIK *in vitro* expansion from health volunteers with informed consent. Peripheral blood mononuclear cells (PBMC) were separated by Ficoll density gradient centrifugation (GE Healthcare) and seeded at a concentration of 1.5×10^6^ cells/ml in culture medium (Gibco, Life Technologies) with the addition of 1000 U/ml interferon-γ (Pepro Tech) and 50ng/ml anti-human OKT-3 (eBioscience) on day 0 and 300U/ml interleukin (IL)-2 (Huaxin High Biotechnology, Shanghai) on day 1. Phenotype and immune profile of CIK cells were characterized by multiparameter flow cytometric analysis using following monoclonal antibodies: CD3-FITC, CD56-PC7, CD3-PC7, CD4-FITC, CD8-FITC, CD314-PC7 (anti-NKG2D), CD152-PE (anti-CTLA-4), CD223-APC (anti-LAG-3), CD226-PE (anti-DNAM-1), CD244-FITC (anti-2B4), CD274-PE (anti-PD-L1), CD279-APC (anti-PD-1), Foxp3-PE (mAbs were from BD Biosciences). Flow cytometric analysis was performed using the Coulter FC500 flow cytometer (Beckman Coulter). Data were further analyzed by Flow Jo 7.6.5 software (Tree Star).

### Tumor cell lines

HGC27, MGC803 and SGC7901 gastric cancer (GC) cell lines as well as SW480, HT-29, RKO, and HCT116 colorectal cancer (CRC) cell lines were obtained from Cell Bank of Type Culture Collection of Chinese Academy of Sciences (Shanghai, China). MKN-45 GC cells were obtained from 3DBiopharm Biotech Co. Ltd. (Shanghai, China). SNU-216 GC cells were from Medical College of Xiamen University (Fujian, China). Cells used in this study were grown in Minimum Essential Medium (HGC-27), or in RPMI-1640 medium (rest of the cells) supplemented with 10% fetal bovine serum (FBS) (Sigma), and 1% penicillin–streptomycin (Invitrogen), in a humidified 5% CO2 incubator at 37°C, and genotypes of cell lines were confirmed and authenticated by short tandem repeat (STR) DNA profiling analysis.

Selected malignant cells (MGC803, HGC27, RKO, and SW480) were stained with PE or FITC-conjugated mAbs: anti-HLA-ABC-FITC, anti-MIC A/B-PE (BD biosciences), and anti-ULBP2 (abcam).

### Generation and lentiviral delivery of small hairpin RNA (shRNA) and PD-L1 cDNA

The sequence of DNA oligonucleotides used to generate shRNA targeting the open reading frame of PD-L1 mRNA on tumor cells (MGC803 and RKO) were as follows: Oligo-F, 5′-CCGGGCATTTGCTGAACGCATTTACCTCGAGGTAAATGCGTTCAGCAAATGCTTTTTTG-3′;Oligo-R,5′-AATTCAAAAAAGCATTTGCTGAACGCATTTACCTCGAGGTAAATGCGTTCAGCAAATGC-3′; The DNA oligonucleotides used to generate scrambled shRNA were 5′-TTCTCCGAACGTGTCACGT-3′. Oligonucleotides were annealed in a buffer containing 100 mM Tris-HCl (pH 7.5) and 20 mM MgCl_2_ for 10 minutes at 95°C, followed by 20 minutes at 65°C. The annealed DNAs were ligated into pLKD-CMV-EGFP-U6-shRNA vector that had been cut with ApaI, blunted with the T4 DNA Ligase, and digested with EcoRI to generate plasmid pLKD/PD-L1i/puromycin. Then the plasmid was transfected into 293T packaging cells to generate lentiviruses, which were used to infect selected tumor cell lines. Negative Controls were generated by infection with viruses containing the scrambled shRNA following the same protocol. In addition, for preparation of lentiviruses with PD-L1 cDNA, 2μg expression plasmid with or without PD-L1 cDNA was transfected into 293T packaging cells using the FuGENE HD Transfection (Roche) according to the manufacture protocol.

All selected tumor cells were plated at a density of 3×10^5^ in six-well plates in advance for transfection in culture medium. About 48h after transfection, the medium was changed and puromycin was added to select stable transfection. Puromycin-resistant colonies were selected in medium and expanded. Successful gene modification via lentiviral delivery was confirmed by qRT-PCR and flow cytometry.

### Detection of apoptosis

Cell apoptosis was detected using the Annexin V-PE Apoptosis Detection Kit (BD Bioscience) according to the manufacturer's instructions. CIK cells was pre-incubated with the tumor cells in 2ml culture medium per well in a 6-well-plate at a E:T ratio of 10:1 overnight, and then both suspending and adherent cells were harvested and stained with Annexin V-PE and 7-AAD as well as CD3 that precludes CIK cells by gate setting, and analyzed on the flow cytometer.

### Functional assays and co-cultures of CIK cells

The cytotoxic activity of CIK cells was assessed by LDH release assay using the CytoTox 96® Non-Radioactive Cytotoxicity Assay (Promega). Briefly, effector CIK cells for each treatment groups were respectively cultured with 5×10^3^ MGC803 or 10^4^ RKO, HGC27, SW480 cells/well (the optimal number for each tumor cell was determined before the assay according to the manufacturer's instructions) in triplicate at varying Effector/Target ratios (40:1, 20:1, 10:1, 5:1) and incubated at 37°C for 4 to 6 hours. The percent cytotoxicity was calculated according to the formula: (experimental release–effector spontaneous release–target spontaneous release)/(target maximum release–target spontaneous release) ×100%. Each treatment group was represented by differential neutralizing antibody against PD-1, PD-L1 (20μg/ml, R&D Systems), NKG2D (20μg/ml, R&D Systems), or ULBP-2 (10μg/ml, abcam) and corresponding isotype antibodies (R&D Systems) added at co-culture initiation.

For the detection of functionally responsive immune receptors associated with co-incubation of CIK and tumor cells, the CIK cells expanded on day 14 were cultured with 5×10^5^ tumor cells (MGC803) at a ratio of 5:1 overnight. FITC-conjugated anti-human-CD8, APC-conjugated anti-human-PD-1, PE-conjugated anti-human-PD-L1, PC7-conjugated anti-human-NKG2D, PE-conjugated anti-human-CTLA-4, PE-conjugated anti-human-DNAM-1, APC-conjugated anti-human-LAG-3, FITC-conjugated anti-human-2B4, and APC-conjugated anti-human-CD107a (BD Biosciences) were used for the surface staining. As for the intracellular staining of CIK cells, phytohemagluttinin (Life Technologies) was added at 1% by volume for re-stimulation and brefeldin A (10 μg/ml, Sigma) was added to the co-culture for the last 4 hours to prevent secretion of the intracellular protein. Briefly, the cells were harvested, washed, surface stained, fixed, and permeabilized using a Cytotofix/Cytoperm kit as instructed by the manufacturer (BD Bioscience), and later were stained with PE-conjugated anti-human-IFN-γ (BD Bioscience), APC-conjugated anti-human-granzyme B (BD Bioscience), and PE-conjugated anti-human-Foxp3 (BD Bioscience), and then analyzed on a FACScalibur flow cytometer (Abs were from BD Biosciences). Corresponding isotype controls were used to define background staining (BD Bioscience).

### Assessment of *in vivo* tumor-killing activity of CIK cells

Three-week-old nude female mice were subcutaneously implanted with an 8mm^3^ tumor fragment prepared beforehand with MGC803 cells injected into five mice. Three days after tumor implantation, mice received weekly intravenous infusions of 10^7^ CIK cells suspended in 200ul PBS. In combined treatment group, CIK cells were pre-treated with anti-PD-1 antibody (30μg/ml) for PD-1 blockade before mice infusions. Mice injected with only PBS were used as untreated controls. Tumor growth was monitored twice a week with a caliper and volume calculated according to the formula: V=4/3×π× (a^2^/2) × (b/2). On day 42, the mice were euthanized. In an independent survival experiment, similar treatment groups were followed daily for survival. Survival curves were analyzed using Prism software. Recovered tumor was fixed overnight in 4% paraformaldehyde, dehydrated, paraffin-embedded, sectioned (5 μm). Immunohistochemical staining was conducted with human anti-CD5 and anti-human Ki-67 antibodies (Sungenebiotech, China).

### Cytokine release assay

Cytokine secretion by CIK cells was assayed by incubating 5×10^5^ CIK cells with 1×10^5^ tumor cells in 500 μl culture medium per well in a 48-well-plate in the presence of anti-PD-L1 or anti-PD-1 (20μg/ml) along with the isotype antibody (20μg/ml) as controls which were separately added into each well. After 48 hours, the supernatants were collected for the measurement of IFN-γ cytokines by ELISA (NeoBioscience, China).

### Tumor sample collection and real-time (RT)-PCR

After institutional review board approval, paired and frozen cancer tissues and adjacent non-malignant tissues from the same individual were collected in 136 stage I to IV GC patients between October 2007 and February 2010 at the Fudan University Shanghai Cancer Center (Shanghai, China) after gastric resection with no prior chemo- or radiotherapy. All matched samples were examined by a pathologist to confirm the presence of tumor or normal tissues.

The total RNA of the above cell lines and patient tissue samples were extracted using Trizol reagent (Invitrogen). Reverse transcription reactions containing 1 μg total RNA were performed with SuperScript II reverse transcriptase (Invitrogen). Real-time quantitative polymerase chain reaction (RT-qPCR) was performed in an Applied Biosystems 7900 Detection system with SYBR Green (Takara). mRNA values were represented by calculating ΔCt, where ΔCt indicates the difference in the threshold cycle between β-actin and target genes. The primers for RT-qPCR analysis are listed in Supplementary Table S1.

### Statistical analysis

Statistical analyses were performed with GraphPad Prism version 5.0 and SPSS 12.0 software. The comparison of cytokine secretion and receptor expression between groups was analyzed by using a Student's t test or one-way ANOVA with Bonferroni post-test. A paired t test was used to compare the differences between data from tumor tissues and matched adjacent normal tissues. All comparisons with P < 0.05 are considered statistically significant.

## SUPPLEMENTARY FIGURES AND TABLE


