# Supplementary material for: Implication of combined PD-L1/PD-1 blockade with cytokine-induced killer cells as a synergistic immunotherapy for gastrointestinal cancer
Source: Oncotarget. 2016 Feb 8;7(9):10332–44. doi: 10.18632/oncotarget.7243 (PMC4891123; doi:10.18632/oncotarget.7243)
Supplement: Supplementary file 1 [file oncotarget-07-10332-s001.pdf]

SUPPLEMENTARY FIGURES AND TABLE

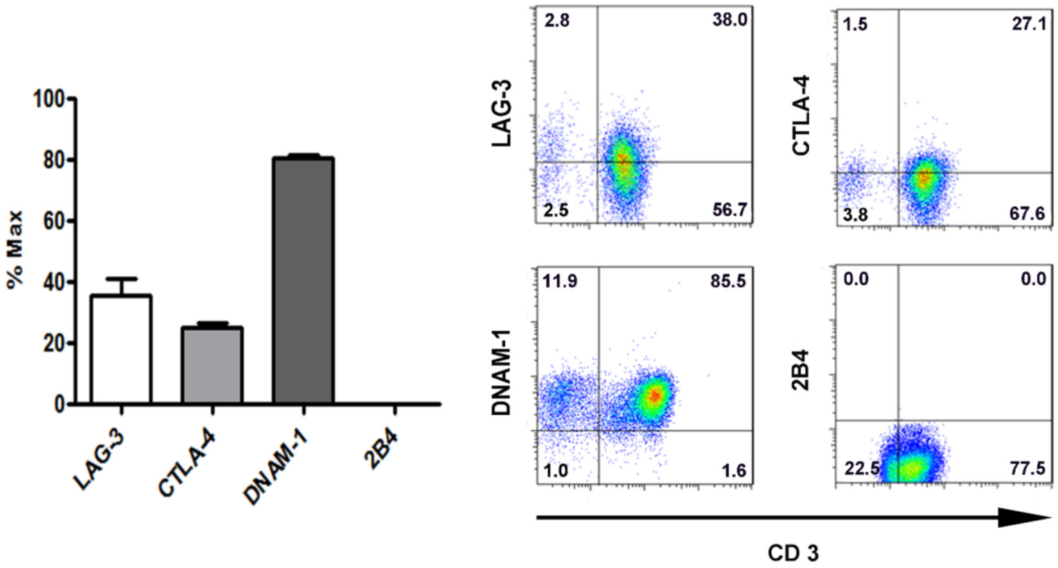

Supplementary Figure S1: Observations on immune-associated receptors on the CIK cells. Percentages of CIK activating receptor, DNAM-1, and inhibitory markers, LAG-3, CTLA-4, and 2B4 were depicted from flow cytometric analysis on day 14, and representative images were shown on the right panel (*n* = 6).

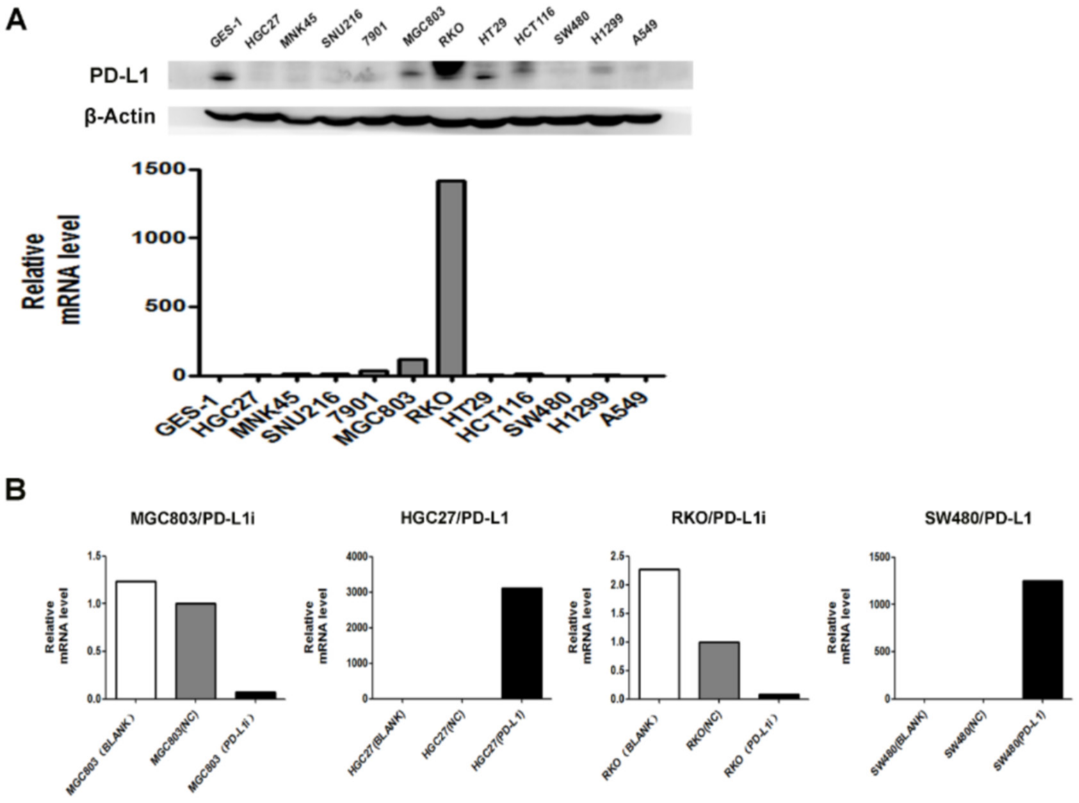

Supplementary Figure S2: Screen of cancer cell lines for their constitutive expression levels of PD-L1. A. Gastric cancer cell lines (GES-1, HGC27, MNK45, SNU216, SGC7901, MGC803) and colorectal cancer cells (SW480, HT-29, RKO, HCT116) were screened at the mRNA and protein levels for their constitutive expression of PD-L1. **B.** MGC803 and RKO with the highest PD-L1 levels in each panel were respectively selected and transduced with lentiviral vectors containing siRNA directed against PD-L1, whereas HGC27 and SW480 with lowest levels were transfected with PD-L1 cDNA. RT-PCR analysis was performed to confirm their expressional variations at the mRNA level.

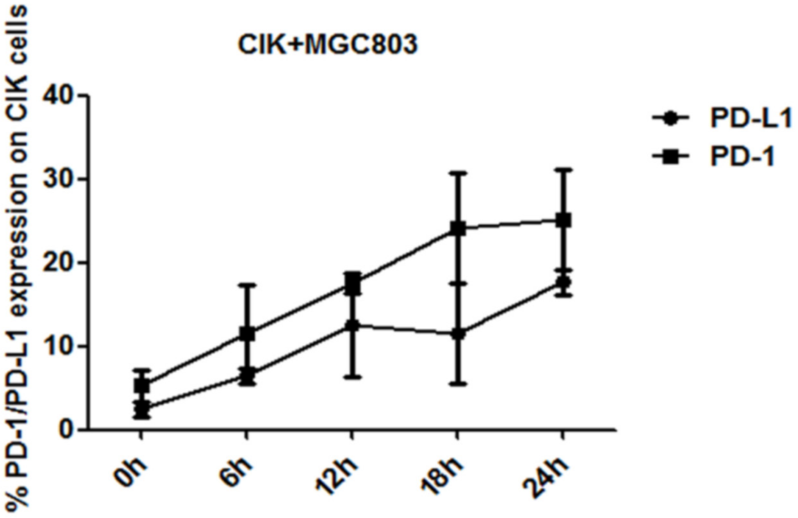

Supplementary Figure S3: Increased levels of PD-1 and PD-L1 on CIK cells over 24 hour-co-culture with MGC803. Upon the co-incubation of CIK cells with MGC803, the changes of PD-1 and PD-L1 levels on CIK cells were examined over 24 hours. Results represent at least two independent experiments using the CIK cells from different donors and are shown as Mean±SEM.

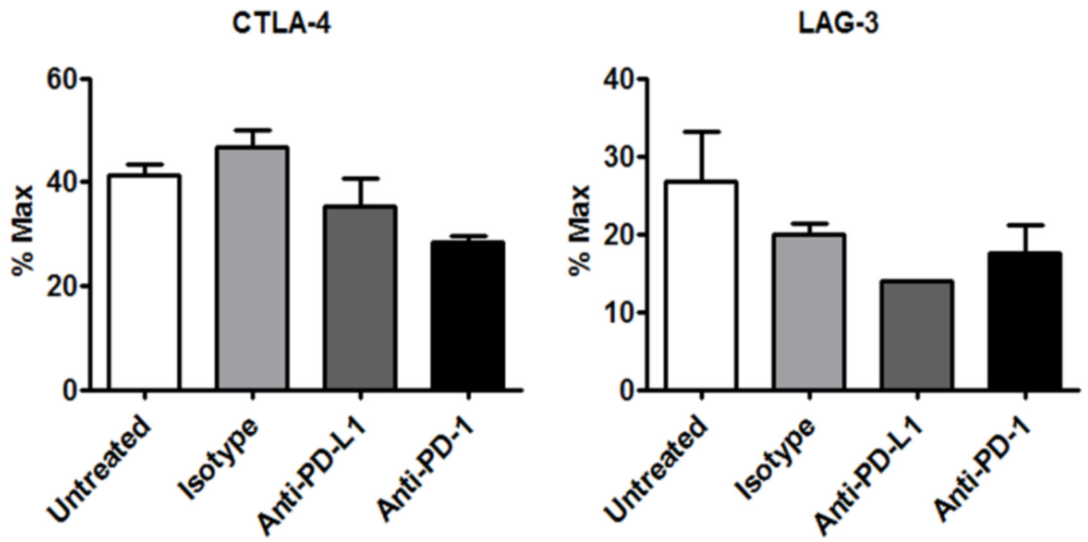

Supplementary Figure S4: Blockade of PD-L1/PD-1 pathway could impair the inhibitory signaling of CIK mediated by CTLA-4 and LAG-3. Associated immune-inhibitory molecules, CTLA-4 and LAG-3, were examined after PD-L1/PD-1 pathway blockade using anti-PD-L1 or anti-PD-1 (20 ug/mL). Results represent at least two independent experiments using the CIK cells from the same donor and are shown as Mean±SEM.

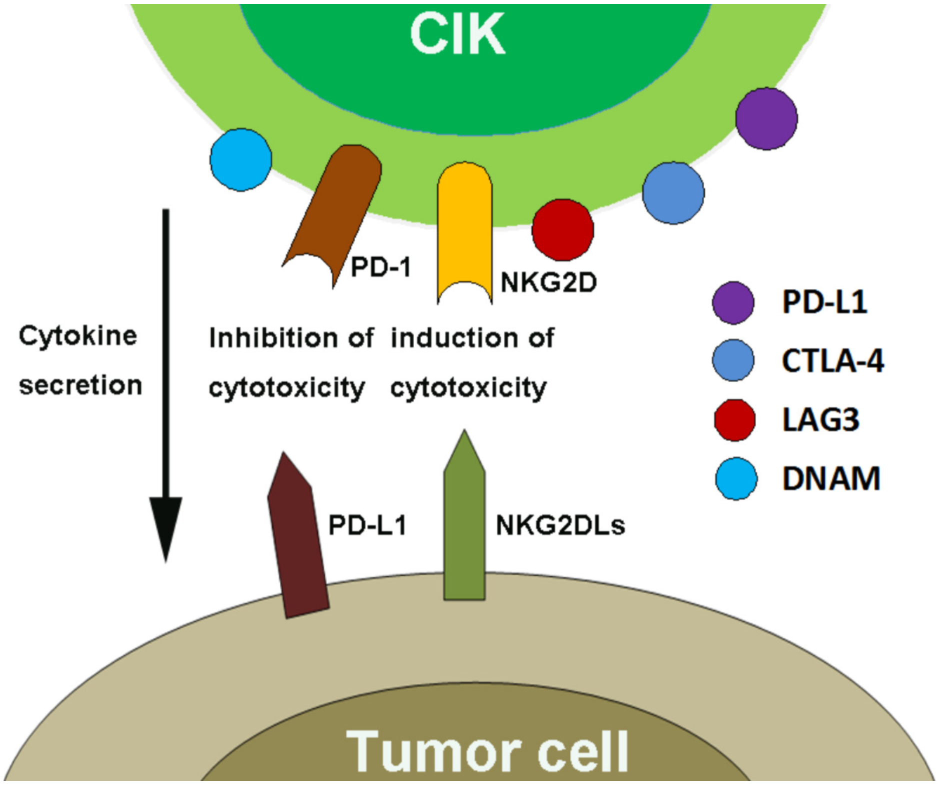

Supplementary Figure S5: The relationship between CIK cells and tumor cells. The diagram depicts that CIK cells kill the tumor cells in an MHC-unrestricted mechanism, through binding of NKG2D receptor with its ligands, while PD-L1/PD-1 inhibits the cytotoxic activity of CIK cells. Other potential functional receptors on the CIK cells might also be involved in the CIK anti-tumor immunity and remain to be explored in the future study.

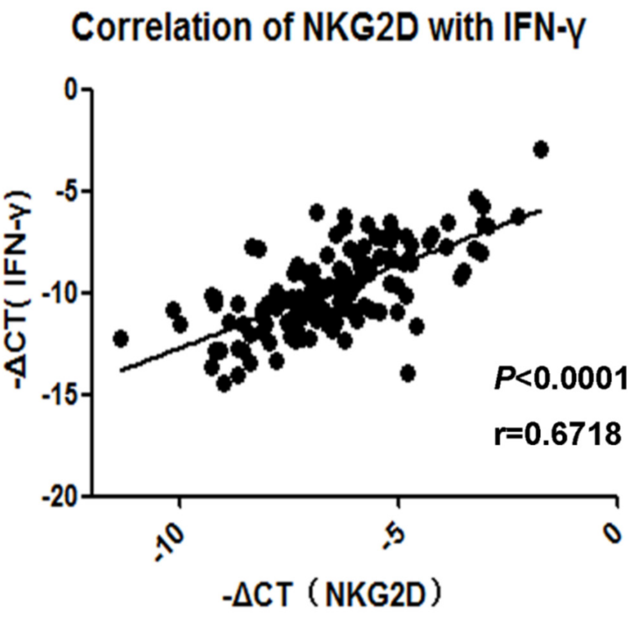

Supplementary Figure S6: NKG2D levels positively correlated with IFN-γ in gastric cancer tissue. Correlations between NKG2D mRNA expression and IFN-γ levels were detected in gastric cancer tissues. Pearson's r and *P* value are displayed. -ΔCt indicates the difference in the threshold cycle between the target genes and β-actin.

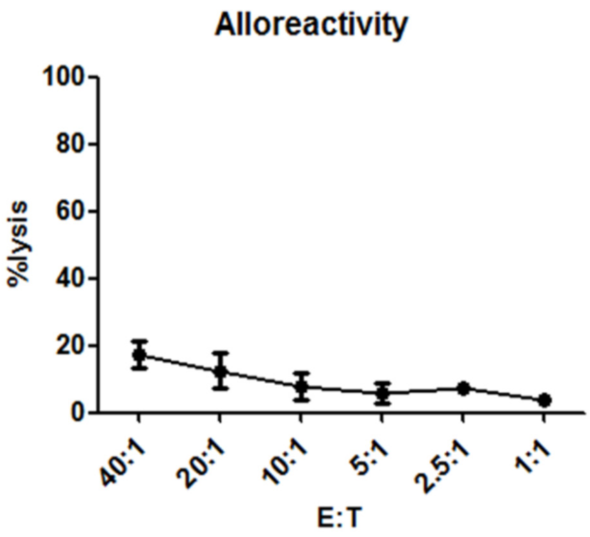

Supplementary Figure S7: CIK cells showed a low alloreactivity against allogeneic peripheral blood mononuclear cells. Alloreactivity of CIK cells was evaluated at different E:T ratios against allogeneic PBMCs by a non-radioactive cytotoxicity assay. Results represent at least two independent experiments using the CIK cells from different donors and are shown as Mean±SEM.

Supplementary Table S1: Primers for qRT-PCR analysis

primer name	primer sequence (5′-3′)
PDL1-F	TGGCATTTGCTGAACGCATTT
PDL1-R	TGCAGCCAGGTCTAATTGTTTT
IFN-r-F	GAGTGTGGAGACCATCAAGGAAG
IFN-r-R	TGCTTTGCGTTGGACATTCAAGTC
NKG2D-F	GGTATGAGAGCCAGGCTTCTTG
NKG2D-R	GAATGGAGCCATCTTCCCACTG
LAG-3-F	GCAGTGTACTTCACAGAGCTGTC
LAG-3-R	AAGCCAAAGGCTCCAGTCACCA
CTLA4-F	ACGGGACTCTACATCTGCAAGG
CTLA4-R	GGAGGAAGTCAGAATCTGGGCA
β-actin-F	GATCTTCGGCACCCAGCACAATGAAGATC
β-actin-R	AAGTCATAGTCCGCCTAGAAGCAT
